# Supplementary material for: Functional Characterization of Ubiquitination Genes in the Interaction of Soybean—Heterodera glycines
Source: Int J Mol Sci. 2022 Sep 15;23(18):10771. doi: 10.3390/ijms231810771 (PMC9504373; doi:10.3390/ijms231810771)
Supplement: Supplementary file 1 [file ijms-23-10771-s001.zip › ijms-1870167-supplementary.pdf]

Supplementary Materials

Table S1. Domain classification of 49 PUB proteins in soybean

Group	Domain organization（N-terminus/C-terminus）	Number of Proteins included
I	UFD2 specific motif + U-box	0
II	U-box+ARM	21
III	U-box + GKL-box	5
IV	U-box + Kinase	5
V	U-box only	16
VI	U-box+WD40	1
VII	U-box+TPR	1
VIII	U-box+MIF4G	0

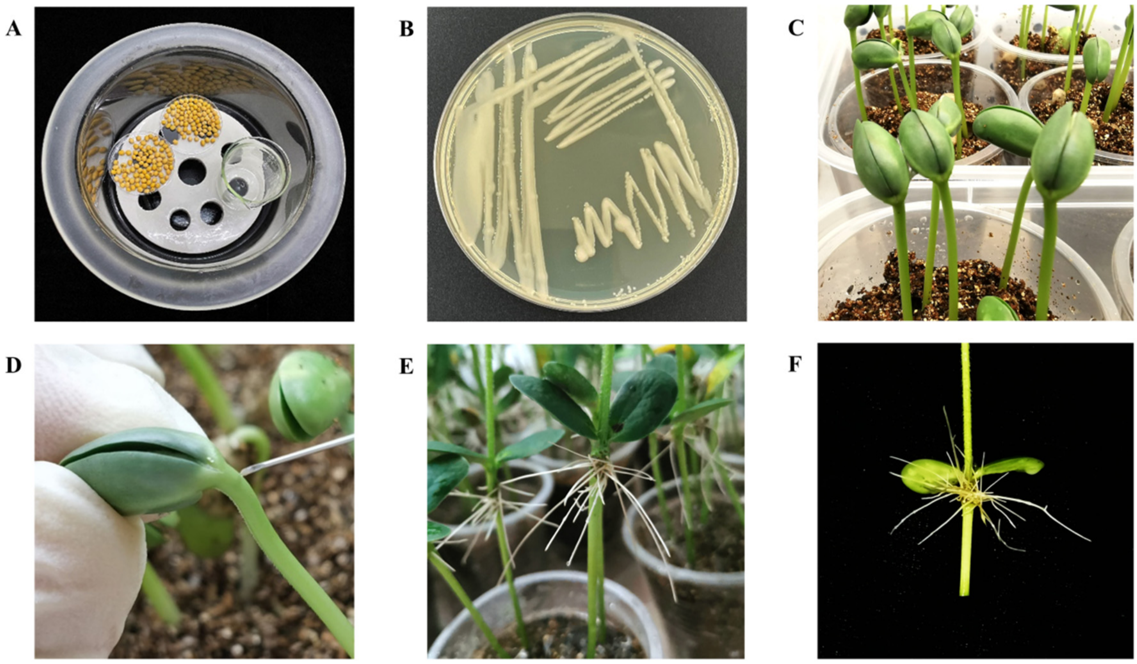

Figure S1. Transformation process of soybean hairy roots. A, Seed sterilization; B, *Agrobacterium rhyzogenes* K599; C, unexpanded soybean cotyledons; D, Soybean W82 seedlings inoculated with *A. rhizogenes K599*; E, Hairy roots generated from infection sites; F, Remove main roots.

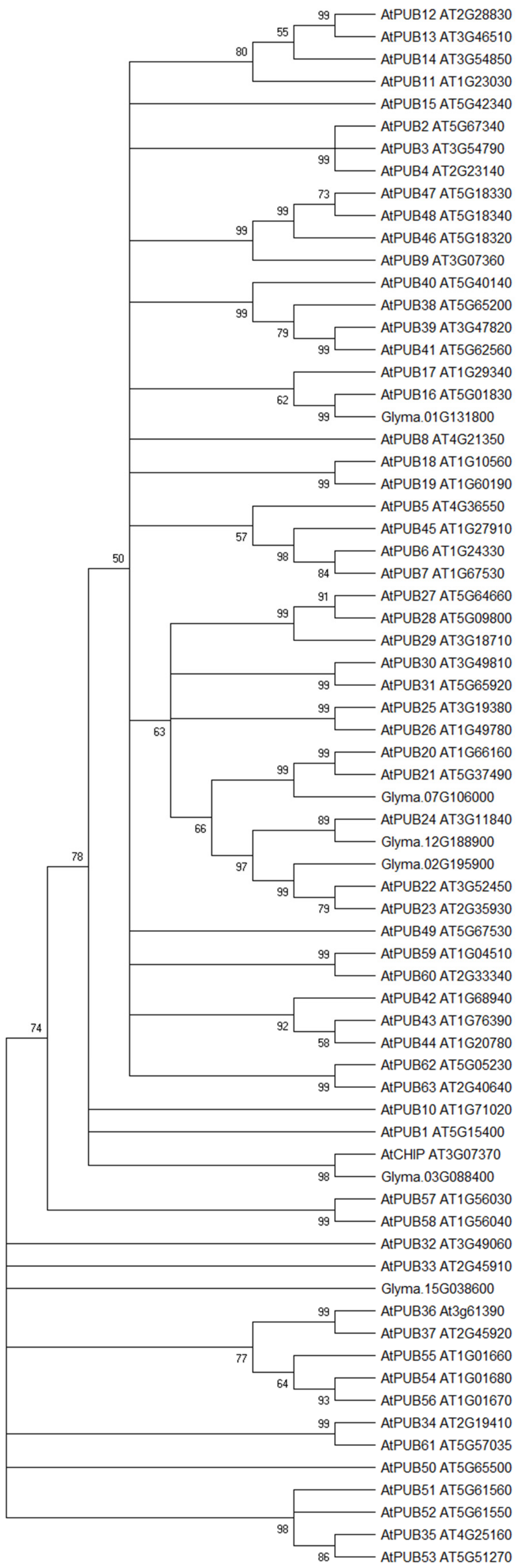

Figure S2. Phylogenetic tree of 64 AtPUBs and 6 GmPUBs based on full-length protein sequences.

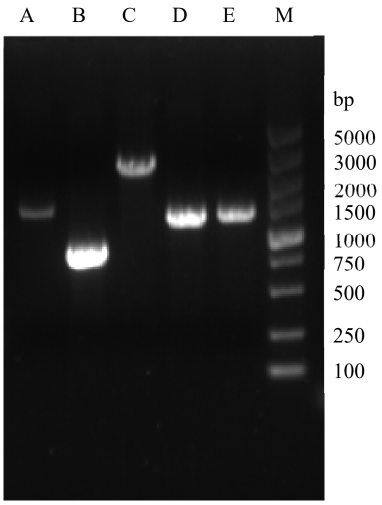

Figure S3. PCR amplified products of five *GmPUBs*. A: *GmPUB20A*; B: *GmCHIPA*; C: *GmPUB33A*; D: *GmPUB23A*; E: *GmPUB24A*;M: DL2000 marker.

```
AtPUB18           KDHDLIRGLKVEDLLCPISLEIMTDPVVIETGHTYDRSSI   318
AtPUB22           ......EIEIPSFFLCPISLDIMKDPVIVSTGITYDRESI    37
AtPUB24           ....EEEIEIPNYFICPISLEIMKDPVTTVSGITYDRQNI    40
Glyma.01G131800   RRNQSLELTIPADYRCPISLELMRDPVVVATGQTYDRASI   303
Glyma.07G106000   ...SITELVIPNHFRCPISLDLMKDPVTLSTGITYDRESV    57
Glyma.03G088400   AAEADTPTEVPDYLCCRITLDIFHDPVITPSGLTYERAVI   231
Glyma.15G038600   ........PVPSFFSCQILLEIMHDPQVAADGFTYEGDAI    60
Glyma.02G195900   ......EIDVPPFFVCPISLELMKDPVTVSTGITYDRDSI    36
Glyma.12G188900   ......EIEIPQFFLCPISLQIMKDPVTTVTGITYDRESI    44
CaPUB1            ......EIQVPPYFLCPISLEMMKDPVTISTGITYDRENI    36
OsPUB15           QLLSINGVPIPADFCCPLSLELMSDPVIVASGQTYERVYI   259
TcPUB23           ......EIDVPPFFICPISLEIMKDPVTVSTGITYDRESI    36
Consensus               ei ip  f cpisleimkdpvt  tgitydr si

AtPUB18           TKWFGSG..NITCPITGKILTST..ELVDNVSVRQVIRKH   354
AtPUB22           EKWLFSG.KKNSCPVTKQVITET..DLTPNHTLRRLIQSW    74
AtPUB24           VKWLEKV...PSCPVTKQPLPLDS.DLTPNHMLRRLIQHW    76
Glyma.01G131800   KLWMDSG..HNTCPKTGQTLSHT..ELIPNRVLRNMIAAW   339
Glyma.07G106000   ERWFDEG..NITCPVTNQVVRN..FDMIPNHSLRIMIQDW    93
Glyma.03G088400   LEHLQKVGK..FDPITREPLDP..SQLVPNLAIKEAVEAF   267
Glyma.15G038600   REWLENG..HDTSPMTNLKLSHL..FLTPNHALRLAIQDW    96
Glyma.02G195900   EKWLFAEVKNDTCPVTKQPLLP...DLTPNHTLRRLIQAW    73
Glyma.12G188900   EKWLLKA.KDCTCPITKQPLPRSPEFLTPNHTLRRLIQAW    83
CaPUB1            ERWIFSA.KNNTCPVTKQSLTSI..ELTPNVTLRRFIQSW    73
OsPUB15           KLWLDEG..FTICPKTRQRLGHS..NLIPNYTVKALIANW   295
TcPUB23           EKWLFSG.KNTTCPVTKQVITDC..DLTPNHTLRRLIQSW    73
Consensus         ekwl  g k  tcpvtkq l      ltpnhtlrrliq w

AtPUB18           CKTNGIVLAGISRRRKSHDDVVPESLAAKGAGKLIAKFLT   394
AtPUB22           CTLN..ASYGIERIPTPKPPICKSEIEKLIKESSSSHLN.   111
AtPUB24           CVEN..ETRGVVRISTPRVPPGKLNVVEEIKNLKKFGQEA   114
Glyma.01G131800   CREQ..RIPFKVETVTGKHNSGVTNKAALEATRMMVSFLV   377
Glyma.07G106000   CVEN..RQHGVERIPTPRIPISPNEVAELLMQVKASARG.   130
Glyma.03G088400   LDKHGWAYKIE.............................   278
Glyma.15G038600   LCKP....................................   100
Glyma.02G195900   CTVN..ASHGVQRIPTPKPPVDKTLIEKLLRNTSASDSPS   111
Glyma.12G188900   CSAN..EANGVDQIPTPKSPLSNSNAEKLVKDLEVSSR..   119
CaPUB1            CTLN..ASHGIERFPTPKPPVSKAQILKLMKEAKSREM..   109
OsPUB15           CESH..NIRLPDPMKSLKLNFPLAASALQDSSTTGSSPLH   333
TcPUB23           CMLN..ASHGIERIPTPKPPISKAQITKLLNDATSPHQ..   109
Consensus         c  n     g  riptpk p        l
```

Figure S4. Multiple sequences alignment of U-box proteins of different plants. Note: Navy, pink and blue shading indicate 100%，75%, 50% homology of amino acids, respectively.

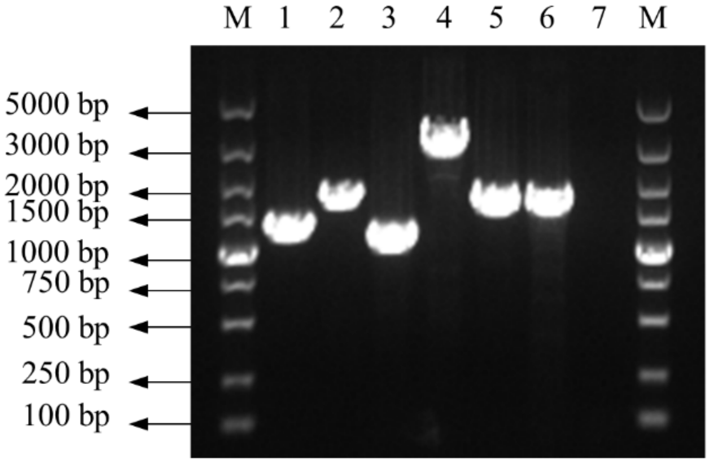

Figure S5. The validation of recombinant overexpressing vectors of five *GmPUB*s by PCR amplification. 1: EV; 2: pOE-GmPUB20A; 3: pOE-GmCHIPA; 4: pOE-GmPUB33A; 5: pOE-GmPUB23A; 6: pOE-GmPUB24A; 7: ddH2O; M: DL5000 marker.

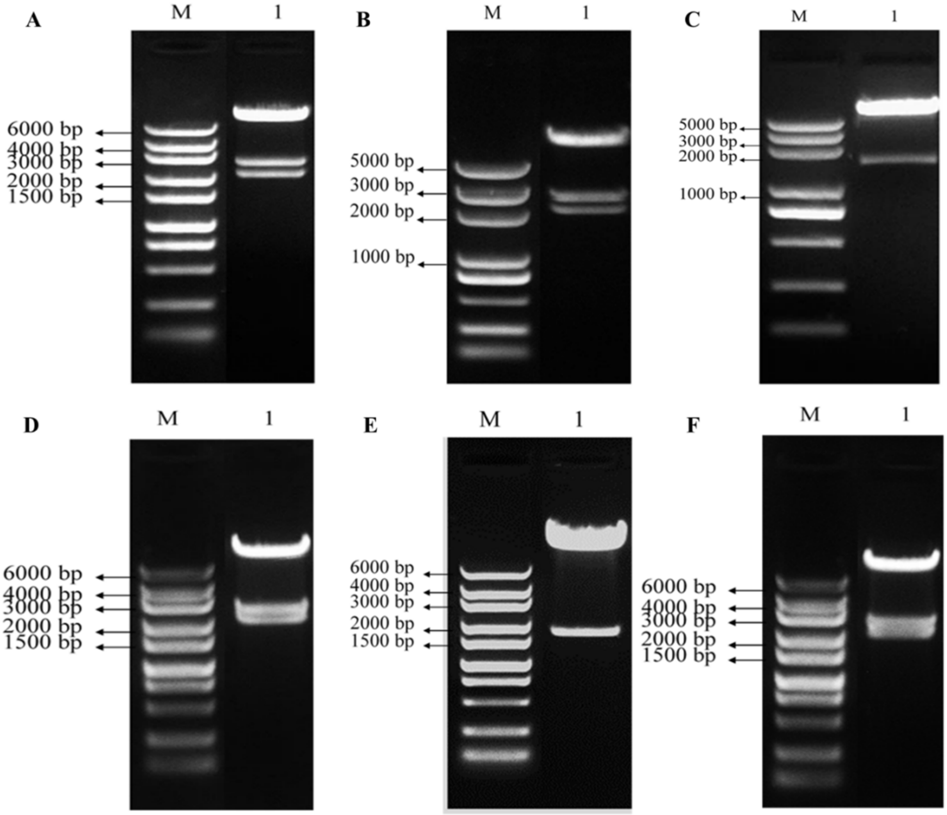

Figure S6. The validation of recombinant RNAi vectors of six *GmPUB*s by enzymes digestion. A: pRNAi-GmPUB16A; B: pRNAi-GmPUB20A; C: pRNAi-GmCHIPA; D: pRNAi-GmPUB33A; E: pRNAi-GmPUB23A; F: pRNAi-GmPUB24A; M: DNA marker.

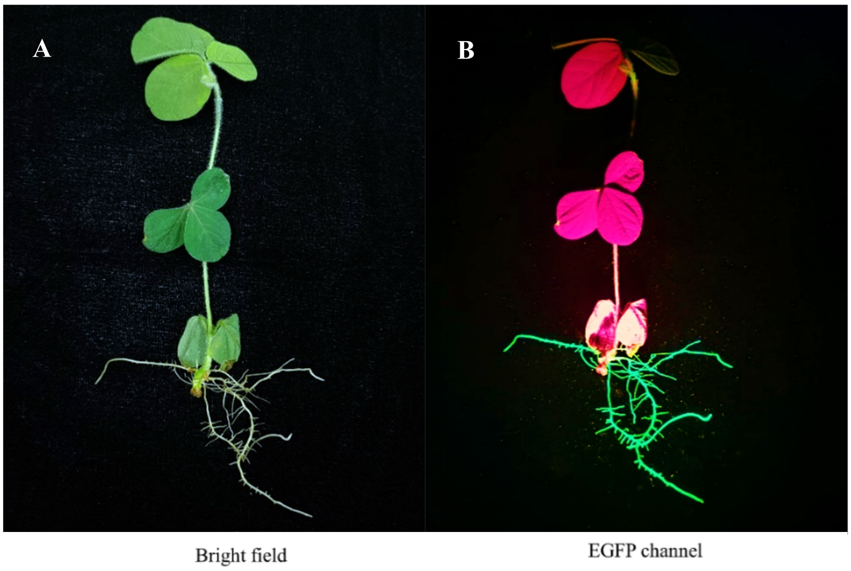

Figure S7. *A. rhizogenes* K599 induces soybean cultivar W82 to produce transgenic hairy roots A, bright field, B, EGFP channel.
